# Diabetic Kidney Disease in FVB/NJ Akita Mice: Temporal Pattern of Kidney Injury and Urinary Nephrin Excretion

**DOI:** 10.1371/journal.pone.0033942

**Published:** 2012-04-04

**Authors:** Jae-Hyung Chang, Seung-Yeol Paik, Lan Mao, William Eisner, Patrick J. Flannery, Liming Wang, Yuping Tang, Natalie Mattocks, Samy Hadjadj, Jean-Michel Goujon, Phillip Ruiz, Susan B. Gurley, Robert F. Spurney

**Affiliations:** 1 Division of Nephrology, Department of Medicine, Duke University and Durham VA Medical Centers, Durham, North Carolina, United States of America; 2 Chung-ang University Medical School, Seoul, Republic of Korea; 3 Division of Cardiology, Department of Medicine, Duke University Medical Center, Durham, North Carolina, United States of America; 4 CHU Poitiers, Pathology Unit, Poitiers, France; 5 INSERM U927, Poitiers, France and INSERM CIC 0802, Poitiers, France and CHU Poitiers, Endocrinologie, Poitiers, France; 6 Department of Surgery and Pathology, University of Miami, Miami, Florida, United States of America; Institut National de la Santé et de la Recherche Médicale, France

## Abstract

Akita mice are a genetic model of type 1 diabetes. In the present studies, we investigated the phenotype of Akita mice on the FVB/NJ background and examined urinary nephrin excretion as a marker of kidney injury. Male Akita mice were compared with non-diabetic controls for functional and structural characteristics of renal and cardiac disease. Podocyte number and apoptosis as well as urinary nephrin excretion were determined in both groups. Male FVB/NJ Akita mice developed sustained hyperglycemia and albuminuria by 4 and 8 weeks of age, respectively. These abnormalities were accompanied by a significant increase in systolic blood pressure in 10-week old Akita mice, which was associated with functional, structural and molecular characteristics of cardiac hypertrophy. By 20 weeks of age, Akita mice developed a 10-fold increase in albuminuria, renal and glomerular hypertrophy and a decrease in the number of podocytes. Mild-to-moderate glomerular mesangial expansion was observed in Akita mice at 30 weeks of age. In 4-week old Akita mice, the onset of hyperglycemia was accompanied by increased podocyte apoptosis and enhanced excretion of nephrin in urine before the development of albuminuria. Urinary nephrin excretion was also significantly increased in albuminuric Akita mice at 16 and 20 weeks of age and correlated with the albumin excretion rate. These data suggest that: 1. FVB/NJ Akita mice have phenotypic characteristics that may be useful for studying the mechanisms of kidney and cardiac injury in diabetes, and 2. Enhanced urinary nephrin excretion is associated with kidney injury in FVB/NJ Akita mice and is detectable early in the disease process.

## Introduction

A significant subset of patients with diabetes mellitus develops diabetic nephropathy (DN) and cardiovascular disease which may result from a constellation of coexistent risk factors including poor glycemic control, albuminuria, hypertension and left ventricular hypertrophy (LVH). In the United States, the incidence of diabetes mellitus has reached epidemic proportions. DN is the most common cause of end-stage renal disease (ESRD) in developed countries [1]. Moreover, in these diabetic patients, the rate of heart disease and stroke is 2 to 4 times higher than in patients without diabetes [2]. Complications of diabetes mellitus are, therefore, a significant healthcare burden. As a result, much effort has been devoted to understanding disease mechanisms in diabetes mellitus with the goal of identifying early markers of diabetic kidney injury as well as new therapeutic targets for the treatment of cardiovascular and kidney disease associated with diabetes.

Mouse models of diabetes mellitus are useful for studying both the pathogenesis and treatment of kidney disease in diabetic humans because of the potential for genetic manipulation [3]. Unfortunately, current mouse models do not display the spectrum of pathological and functional characteristics of human diabetes [3]. In this regard, Akita mice are a mouse model of type 1 diabetes mellitus caused by a spontaneous point mutation in the *Ins2* gene [3], [4], [5], [6]. The renal phenotype of these mice is significantly influenced by genetic background [3], [4], [5]. Most studies, however, have utilized Akita mice on the C57BL/6 background and these mice develop only mild kidney disease including modest levels of albuminuria and renal pathological changes limited to mesangial expansion [4]. In the present studies, we investigated the phenotype of Akita mice on a distinct genetic background: FVB/NJ. This strain is frequently used to create genetically modified mice because these animals are vigorous breeders with large litters and have prominent pronuclei, which facilitate pronuclear injections when creating transgenic animals [7]. Thus, Akita mice on the FVB/NJ background might offer advantages in studies using genetic approaches.

Glomerular podocytes are highly differentiated cells that play a pivotal role in maintaining the integrity of the glomerular filtration barrier [8], [9]. In both humans and animals with diabetic kidney disease, the number of glomerular podocytes is reduced [10], [11], [12], [13], [14], [15]. While the causes of podocyte loss in DN are likely multifactorial [15], [16], apoptosis plays a prominent role [13], [15], [17]. Because podocytes have limited replicative potential [8], [16], a sufficient loss of podocytes leads to instability of the glomerular tuft, alterations in the integrity of the glomerular filtration barrier and eventually glomerulosclerosis [16]. An important component of the glomerular filtration barrier is the podocyte protein nephrin [9], [18], [19]. In diabetic kidney disease, alterations in glomerular expression of nephrin may contribute to a decrease in the integrity of the glomerular filtration barrier [20], [21], [22], [23], [24]. Moreover, the nephrin protein can be detected in urine of both animals [23] as well as patients with diabetes mellitus [21], suggesting that it may be a marker of kidney injury in diabetes [5].

In the present studies, we determined the functional and structural characteristics of cardiac and kidney disease in FVB/NJ Akita mice as well as quantitated podocyte number, podocyte apoptosis and urinary nephrin excretion. The goal of these studies was twofold. First, a detailed phenotype of FVB/NJ Akita mice has not been previously reported. We, therefore, carefully characterized cardiac and kidney disease in this model and compared our findings to published studies in other Akita strains to determine advantages and limitations of the FVB/NJ Akita model. Secondly, we investigated the temporal relationship between urinary nephrin excretion and both albuminuria and podocyte apoptosis to determine if urinary nephrin excretion could be used as a biomarker of podocyte injury early in the disease process. Our results suggest that Akita mice on the FVB/NJ background have phenotypic features that may be useful for studying mechanisms of kidney and cardiac injury in diabetes mellitus, and that enhanced urinary nephrin excretion is associated with kidney injury in FVB/NJ Akita mice and is detectable with the onset of podocyte apoptosis before the development of albuminuria.

## Results

### Sustained hyperglycemia and polyuria in FVB/NJ Akita mice

**Figure 1:**
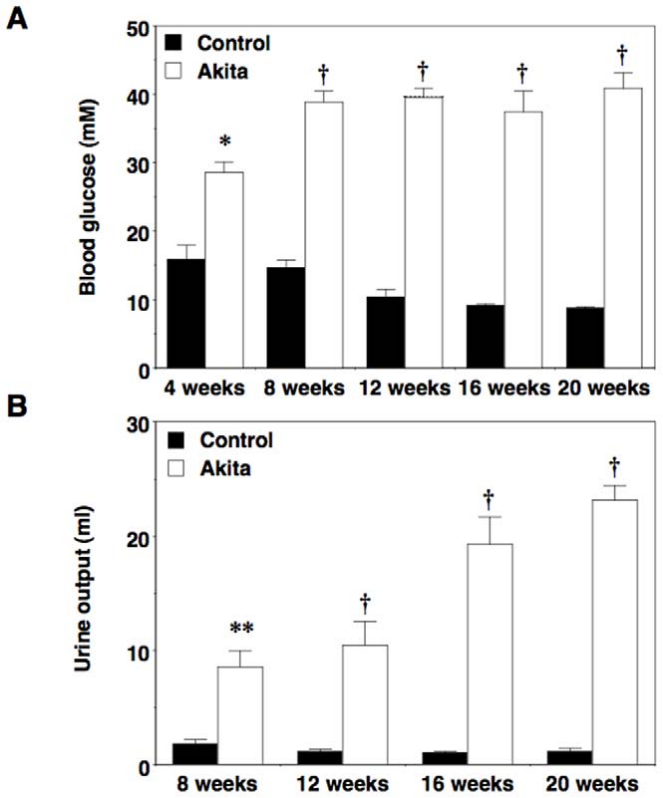
Fasting blood glucose levels and urine output in male Akita mice. (A) FVB/NJ-*Ins2* ^+/C96Y^ mice developed sustained hyperglycemia by 4 weeks of age, which persisted throughout the duration of the study. (B) Urine output in FVB/NJ-*Ins2* ^+/C96Y^ was significantly increased by 8 weeks of age and further increased during the study period. Black bars = control (n = 6 to 8); white bars = Akita (n = 6 to 8). Data are mean ± SEM. *P<0.05 †P<0.001 or **P<0.01 vs. age-matched controls.

As shown in Figure 1A and 1B, male FVB/NJ-*Ins2* ^+/C96Y^ mice (Akita) developed significant hyperglycemia as well as polyuria by 4 and 8 weeks of age, respectively. Fasting blood glucose levels remained elevated in diabetic mice through the 20-week study period. Despite severe hyperglycemia, FVB/NJ-*Ins2* ^+/C96Y^ mice appeared healthy and body weights were similar to non-diabetic controls (Table 1).

**Table 1:** Body weight.

Age (weeks)	Body Weight (g)	
	Control	Diabetic
6	24.1±1.5 (n = 4)	22.5±0.4 (n = 12)
8	24±1.9 (n = 4)	25.8±0.3 (n = 8)
14	30.2±0.9 (n = 8)	30.8±0.2 (n = 8)
16	30.1±0.9 (n = 8)	30.8±0.4 (n = 8)
20	32.1±1.2 (n = 8)	31.9±0.2 (n = 6)

Values are means ± SEM; n, number of mice.

### Urinary albumin excretion (UAE) in diabetic mice

**Figure 2:**
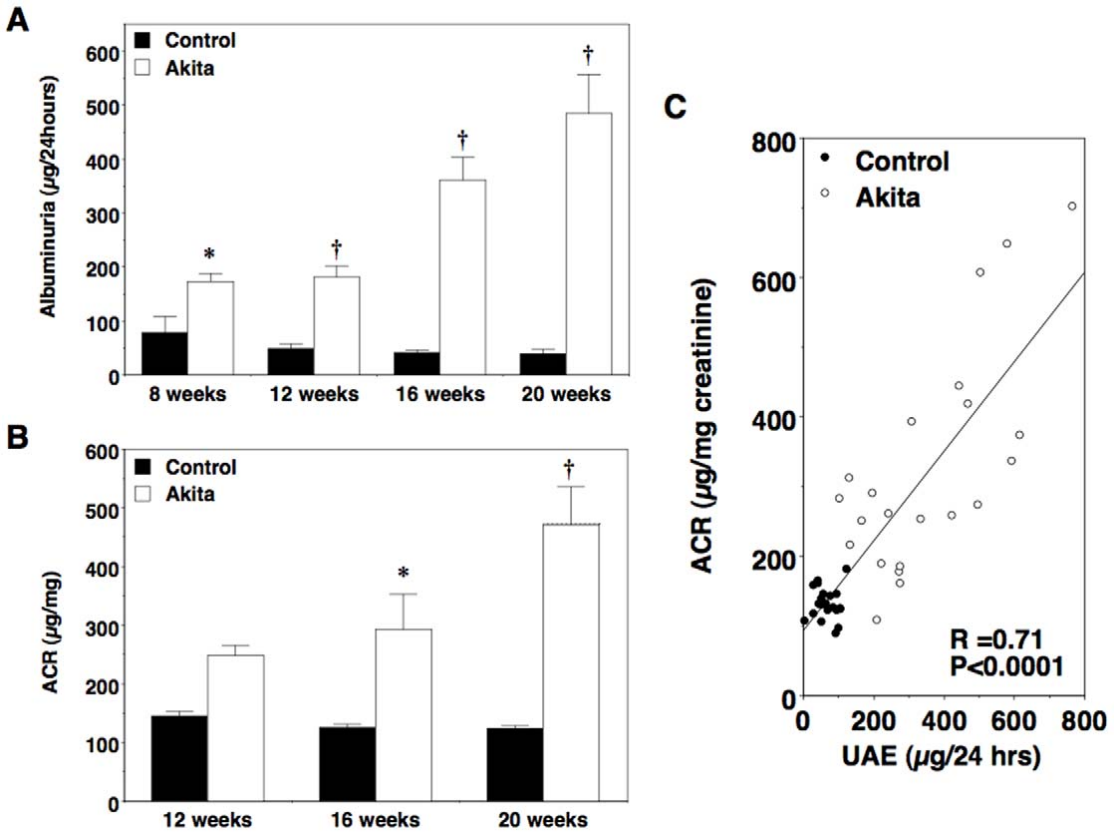
Urinary albumin excretion (UAE) in male Akita mice. (A) UAE was significantly elevated compared with controls in diabetic Akita mice by 8 weeks of age. By 20 weeks of age, there was a >10-fold increase in albuminuria in diabetic mice compared to control animals. (B) Albumin-to-creatinine ratio (ACR) was also significantly increased in diabetic mice compared to age-matched controls. (C) There was a linear relationship between 24-hour UAE and ACR in all mice (R^2^ = 0.71; P<0.0001). Black bars = control (n = 6 to 8); white bars = Akita (n = 6 to 8). Black circles = control; white circles = Akita. Data are mean ± SEM. *P<0.05 or †P<0.0001 vs. age-matched controls.

We measured 24-hour UAE and albumin-to-creatinine ratio (ACR) to assess glomerular injury in Akita mice. As shown in Figure 2A, UAE was significantly increased by 8 weeks of age in diabetic FVB/NJ-*Ins2* ^+/C96Y^ mice compared to their non-diabetic controls and remained elevated over time. At 20 weeks of age, albuminuria was increased by greater than 10-fold in FVB/NJ-*Ins2* ^+/C96Y^ mice compared to their age-matched controls. A similar pattern was observed with ACR (Figure 2B). As shown in Figure 2C, there was a significant linear correlation between 24-hour UAE and ACR for all mice (R^2^ = 0.71; P<0.0001).

### Kidney pathology in FVB/NJ Akita mice

**Figure 3:**
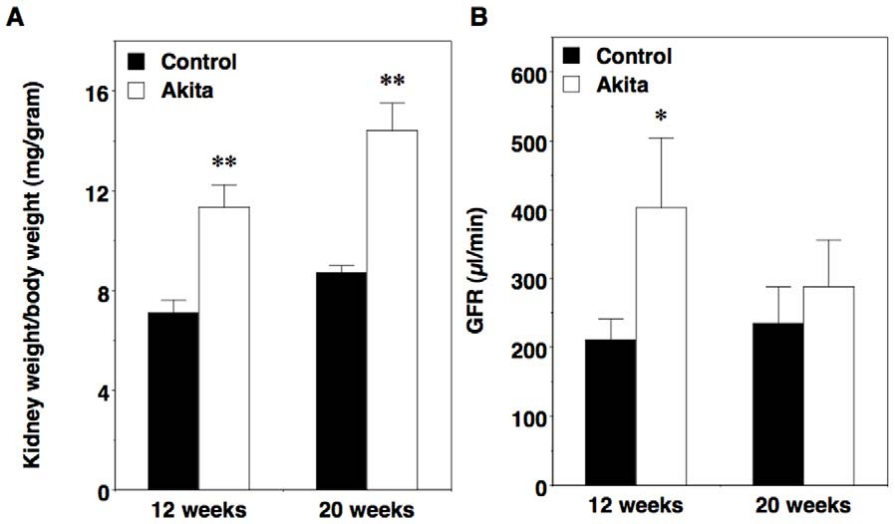
Kidney weight and glomerular filtration rate (GFR) in male Akita mice. (A) Kidney-to-body weight ratio was significantly increased in diabetic Akita mice (n = 6 to 8) compared to controls (n = 6 to 8) at 12 and 20 weeks of age. (B) There was a significant increase in GFR at 12 weeks of age in diabetic Akita mice (n = 3 to 5) compared to controls (n = 5). Black bars = control; white bars = Akita. Data are mean ± SEM. *P<0.05 or **P<0.001 vs. age-matched controls.

**Figure 4:**
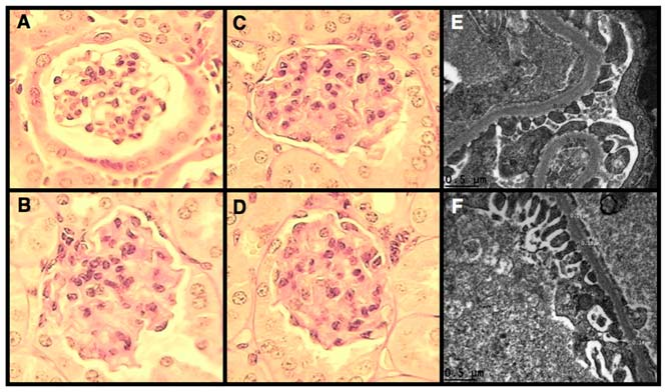
Glomerular histopathology of diabetic Akita mice. Photomicrographs of mouse kidneys were taken using tissue samples prepared from mice at 30-weeks of age. (A) A representative picture from a control mouse (score of 0). (B–D) Moderate mesangial expansion in glomeruli from Akita mice (score of 2). (E) A representative picture of glomerular ultrastructure from a control mouse. (F) FP effacement was not detected by electron microscopy in diabetic Akita mice at 30-weeks of age. Light microscopic sections were stained with Periodic acid-Schiff (PAS) and the magnification was 400×.

As shown in Figure 3A, we found a significant increase in kidney-to-body weight ratio in FVB/NJ-*Ins2* ^+/C96Y^ mice compared to age-matched controls at 12 weeks of age, which was sustained at the 20-week time point. In contrast, kidney-to-body weight ratio was similar in 3-week-old Akita mice and controls (7.3±0.2 [Akita] vs. 7.9±0.3 [control]; P = NS) prior to the development of either sustained hyperglycemia (155±16 mg/dl [Akita] vs. 173±21 mg/dl [control]; P = NS) or enhanced albuminuria (40±7 µg/24 hours [Akita] vs. 31±6 µg/24 hours [control]; P = NS) as previously published [25]. The increase in kidney size in 12-week-old Akita mice was associated with a significant increase in glomerular filtration rate (GFR) compared to controls (Figure 3B). By 20 weeks of age, however, this hyperfiltration was lost. Examination of the kidney by light microscopy revealed an increase in glomerular volume in diabetic mice compared to non-diabetic controls at 20 weeks of age (3.69±0.22×10^5^ µm^3^ [Akita] vs. 2.23±0.13×10^5^ µm^3^ [control]; P<0.001). Mesangial matrix area also tended to be increased in diabetic mice at the 20-week time point; however, this difference did not reach statistical significance (364±20 µm^2^ [Akita] vs. 349±23 µm^2^ [control]; P = NS). A separate group of mice was, therefore, studied at 30 weeks of age. As shown in Table 2, hyperglycemia, albuminuria and GFR were similar to 20-week old Akita mice at the 30-week time point. In contrast to 20-week old Akita mice, however, there was a significant increase in mesangial expansion at 30 weeks of age as depicted in the representative glomeruli shown in Figure 4. The extent of glomerular mesangial expansion was scored using a semiquantiative scale of 0–3 (0-normal, 1-mild, 2-moderate, 3-severe) as described previously [4], [5]. At 30 weeks of age, mesangial score was significantly higher in Akita mice compared to age-matched controls (1.41±0.24 [Akita] vs. 0.00±0.00 [control]; P<0.005). There was also a trend toward an increase in both glomerular basement membrane (GBM) and proximal tubule membrane thickness, but these differences did not reach statistical significance (Table 3). Foot process (FP) effacement was not detected in diabetic Akita mice at 30-weeks of age (Figure 4E and 4F).

**Table 2:** Kidney phenotype at 30 weeks of age.

	30 Weeks	
	Control (n = 6)	Diabetic (n = 4)
Blood Glucose (mmol/l)	11±1	43±2*
Albumin Excretion (µg/day)	52.4±18.4	572.0±197.1*
Glomerular Filtration Rate (µl/min)	373.5±53.0	394.2±51.9

Values are means ± SEM; n, number of mice;

* P<0.05 between diabetic and control animals.

**Table 3:** GBM and proximal tubule basement membrane thickness at 30 weeks of age.

	Basement membrane thickness	
	Control (n = 4)	Diabetic (n = 4)
Glomerular	224.2±27.7 nm	240.8±77.5 nm
Proximal tubule	325.0±47.9 nm	367.5±62.9 nm

Values are means ± SEM; n, number of mice.

### Urinary nephrin excretion and podocyte injury in diabetic mice

**Figure 5:**
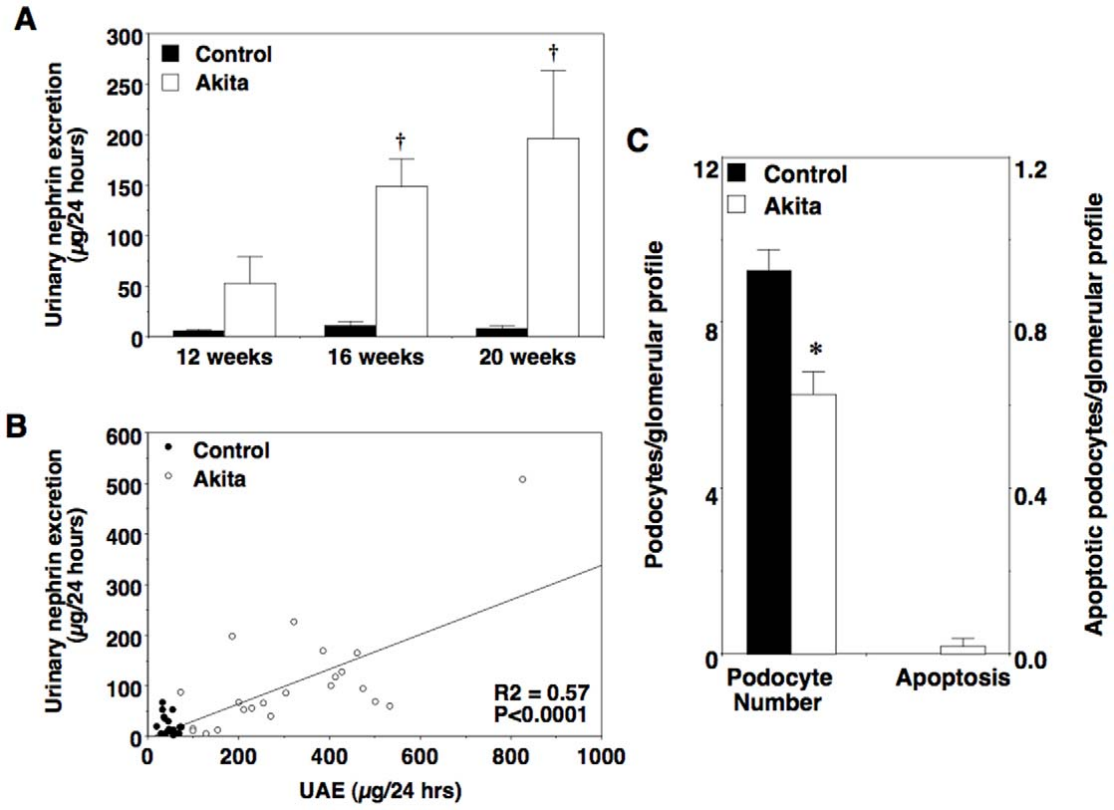
Podocyte injury in albuminuric diabetic Akita mice. (A) Urinary nephrin excretion was significantly enhanced in Akita mice compared to controls at 16 and 20 weeks of age. (B) There was a significant linear relationship between urinary nephrin excretion and albuminuria in all mice (R^2^ = 0.57; P<0.0001). (C) Podocyte number was significantly decreased in Akita mice at the 20-week time point. In contrast, podocyte apoptosis was difficult to detect in either Akita mice or control animals. Black bars = control (n = 6 to 8); white bars = Akita (n = 6 to 8). Black circles = control; white circles = Akita. Data are mean ± SEM. *P<0.05 or †P<0.01 vs. age-matched controls.

As shown in Figure 5A and 5B, excretion of nephrin in urine was increased in diabetic FVB/NJ-*Ins2* ^+/C96Y^ mice at 16 and 20 weeks of age and correlated with albumin excretion rates (R^2^ = 0.57; P<0.0001). To determine if enhanced nephrin excretion was accompanied by histologic evidence of podocyte injury, kidneys were harvested from 20-week old mice and both podocyte number and apoptosis were quantitated as described in the Methods section. Consistent with the results of other investigators [13], podocyte number was significantly decreased in Akita mice compared to controls at 20 weeks of age (Figure 5C). In contrast, podocyte apoptosis was difficult to detect in either Akita mice or control animals at this time point.

**Figure 6:**
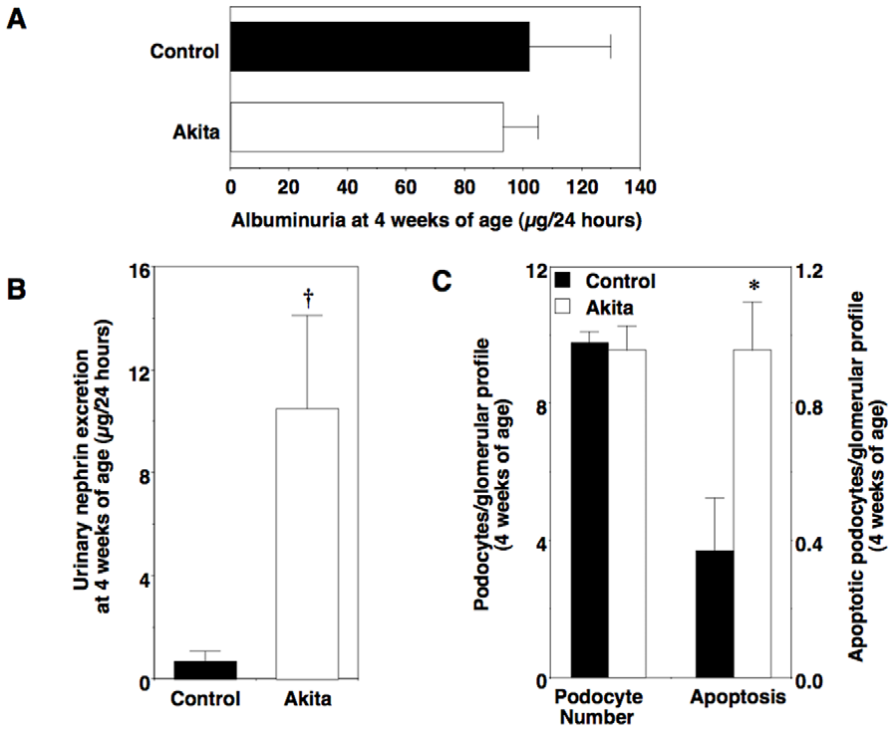
Podocyte injury in normoalbuminuric diabetic Akita mice. (A) Albuminuria was not significantly different in Akita mice and controls at 4 weeks of age. (B) In contrast, urinary nephrin excretion was increased in normoalbuminuric 4-week old Akita mice compared to control animals. (C) There was a significant increase in podocyte apoptosis in Akita mice compared to age-matched controls without a change in podocyte number. Black bars = control (n = 6 to 8); white bars = Akita (n = 6 to 8). Data are mean ± SEM. *P<0.05 or †P<0.001 vs. age-matched controls.

To determine if urinary nephrin excretion was increased in normoalbuminuric Akita mice, we measured excretion of nephrin in urine from 4-week old Akita mice. At this age, Akita mice are hyperglycemic (Figure 1) but albumin excretion rates are similar to age-matched controls (Figure 6A). As shown in Figure 6B, urinary nephrin excretion was significantly enhanced in 4-week old Akita mice compared to control animals. We then harvested kidneys and quantitated both podocyte number and apoptosis (Figure 6C) as described in the Methods section. In contrast to the 20-week time point, podocyte apoptosis was significantly increased in Akita mice compared to control animals with no significant difference in podocyte number. A few apoptotic cells were also detected that did not localize to the glomerular area.

### Cardiovascular pathology in Akita mice

**Figure 7:**
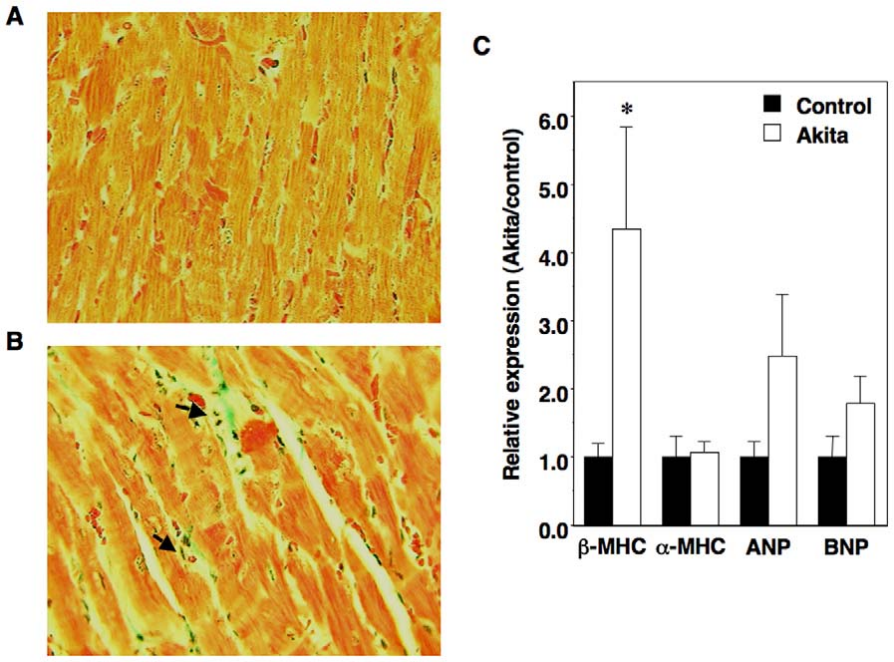
Cardiac injury in male Akita mice. (A–B) Panels A and B show representative photomicrograph of heart sections from controls (A) and diabetic mice (B) at 10 weeks of age. Examination of Akita mice hearts revealed mild interstitial fibrosis (green) and focal inflammatory infiltrates (arrows). Tissue sections were stained with Masson trichrome and the magnification was 400×. (C) Expression of mRNA for β-myosin heavy chain (β-MHC) was enhanced in 10-week old Akita mice. Levels of mRNA were expressed relative to controls (assigned as 1.0) and normalized to β-actin mRNA level. Black bars = control (n = 5); white bars = Akita (n = 5). Data are mean ± SEM. *P<0.05 vs. age-matched controls.

Systolic blood pressure (BP) was significantly elevated in diabetic mice at 10 weeks of age compared to control mice (140±2 mmHg [Akita] vs. 131±2 mmHg [control]; P<0.05). This increase in systolic BP was associated with a significant increase in the ratio of heart-to-body weight in diabetic Akita mice compared to age-matched non-diabetic controls (6.1±0.4 mg/g [Akita] vs. 5.1±0.3 mg/g [control]; P<0.05). Table 4 shows echocardiographic results in mice at 10 weeks of age. Cardiac hypertrophy in Akita mice was associated with a significant decrease in LV fractional shortening consistent with LV systolic dysfunction. There was no statistical difference in interventricular septal width, posterior wall thickness or LV mass by echocardiography (Table 4). Histologic examination of Akita hearts revealed interstitial fibrosis and inflammatory infiltrates in 10-week old mice (Figure 7A and 7B). Analysis of the cardiac pathology using a semiquantitative scoring system found a statistically significant increase in both interstitial fibrosis (0.53±0.23 [Akita] vs. 0.00±0.00 [control]; P<0.005) and inflammatory infiltrates (0.53±0.23 [Akita] vs. 0.00±0.00 [control]; P<0.005) in Akita mice compared to wild-type controls. These pathologic changes were accompanied by enhanced expression of mRNA for the molecular marker of cardiac hypertrophy β-myosin heavy chain (β-MHC) (Figure 7C). Lastly, we investigated expression of cardiac cytokines and markers of fibrosis. As shown in Table 5, there was a significant increase in expression of connective tissue growth factor (CTGF) in Akita mice compared to controls. Transforming growth factor β (TGFβ) and monocyte chemotactic protein-1 (MCP-1) also tended to be increased but these differences did not reach statistical significance.

**Table 4:** Echocardiographic analysis.

	Echocardiographic Analysis	
	Control (n = 5)	Diabetic (n = 5)
LVFS	0.64±0.03	0.56±0.02*
LVDd (mm)	3.85±0.07	3.80±0.09
LVDs (mm)	1.38±0.12	1.67±0.13
IVSW (mm)	0.88±0.04	0.83±0.04
PW (mm)	0.91±0.03	0.87±0.03
LVm (mg)	133.50±7.20	121.20±6.60

Echocardiographic analysis was performed at 10 weeks of age. Values are means ± SEM; n, number of mice; IVSW, interventricular septal width; LVDd, left ventricular end diastolic diameter; LVDs, left ventricular end systolic diameter; LVFS, left ventricular fractional shortening; LVm, left ventricular mass; PW, posterior wall thickness.

* P<0.001 between diabetic and control animals.

**Table 5:** Gene expression in cardiac tissue.

	Cardiac Gene Expression
	Diabetic
TGFβ	1.82±0.40 (n = 5)
MCP-1	2.11±0.84 (n = 5)
IL1β	1.01±.25 (n = 5)
TNFα	0.58±0.16 (n = 5)
CTGF	4.05±0.88 (n = 5)*
Fibronectin	1.54±0.24 (n = 5)

Cardiac gene expression at 10 weeks of age; mRNA levels in diabetic mice relative to controls (assigned as 1.0) normalized to β-actin mRNA level. Values are means ± SE; n, number of mice; TGFβ, transforming growth factorβ; MCP-1, monocyte chemoatactic protein-1; IL1β, interleukin 1β; TNFα, tumor necrosis factor-α; CTGF, connective tissue growth factor.

* P = 0.001 between diabetic and control animals.

## Discussion

DN is the most common cause of ESRD in developed countries [1]. Patients with diabetes are also more likely to develop cardiovascular diseases including stroke and diabetic cardiomyopathy [2]. While BP control and blockade of the renin-angiotensin system can effectively slow disease progression, current therapies cannot prevent progressive kidney and cardiac injury in diabetic patients with established disease [26]. As a result, much effort has been devoted to developing more effective treatments for kidney and cardiovascular diseases in diabetic patients. To achieve this goal, animal models that mimic the human condition would be useful to better understand pathogenic mechanisms as well as to evaluate novel treatment strategies. In this regard, accumulating evidence suggests that glomerular podocytes play a key role in the pathogenesis of diabetic kidney disease [15]. Studies indicate that a decrease in glomerular podocytes is a characteristic feature of both humans and animals with diabetic kidney disease [11], [12], [13], [14], [15], [17]. Because podocytes are terminally differentiated cells with little potential for proliferation [8], [16], podocytes that are lost cannot be effectively replaced which may lead to instability of the glomerular tuft and glomerulosclerosis [16], [27]. We, therefore, speculated that detecting podocyte injury before podocyte loss is sufficient to cause glomerular damage might permit intensification of medical therapies to preserve podocyte number and, in turn, delay or prevent diabetic kidney disease. In this regard, urinary excretion of the slit diaphragm protein nephrin [22], [28], [29] can be detected in diabetic animals at the onset of albuminuria [23] as well as in normo-, micro- and macroalbuminuric patients with diabetes [21], perhaps reflecting early podocyte damage. To further determine if nephrin might be a useful early marker of podocyte injury, we quantitated nephrin excretion in urine of FVB/NJ Akita mice. We found that the onset of hyperglycemia in 4-week old FVB/NJ Akita mice was accompanied by an increase in podocyte apoptosis and enhanced excretion of nephrin in urine before the development of albuminuria. Urinary nephrin excretion was also increased in older albuminuric Akita mice and correlated with the degree of albuminuria. While further studies will be necessary to determine if these findings are both generalizable to diabetic humans and useful for modifying treatment strategies, these data, taken together with other published studies [21], [23], suggest that enhanced urinary nephrin excretion is associated with kidney injury in diabetes and can be detected early in the disease process.

We also characterized the cardiac and kidney phenotype of male FVB/NJ Akita mice and compared our findings to published studies in other Akita strains (Table 6). We focused on male animals because female Akita mice are resistant to the development of severe hyperglycemia [4], and published data on female mice are limited. As shown in Table 6, sustained hyperglycemia was characteristic of male Akita mice on the C56BL/6, DBA/2, 129/SvEv, FVB/NJ and C3H genetic backgrounds. Moreover, most strains developed hypertension as well as cardiac hypertrophy. Hyperglycemia was associated with hyperfiltration, renal hypertrophy and albuminuria in all genetic backgrounds in which published data was available. The albumin excretion rate was, however, variable between strains with FVB/NJ mice developing the most robust albuminuria. In this regard, UAE in diabetic mice was elevated more than 10-fold above controls by 20 weeks of age. This level of albuminuria has been suggested as an important criterion for validating a mouse model of diabetic nephropathy [30]. In contrast to albuminuria, however, the histological findings in the FVB/NJ model were mild and similar to the histological findings reported on other Akita genetic backgrounds. By 30 weeks of age, diabetic FVB/NJ Akita mice developed mild-to-moderate glomerular mesangial expansion as has been reported on the C56BL/6, 129/SvEv and C3H strains. We did not detect more advanced lesions, such as glomerular sclerosis, tubulointerstitial fibrosis, arteriolar hylanosis, GBM thickening or FP effacement and, to our knowledge, these more advanced histopathological lesions have not been reported on other genetic backgrounds. Taken together these data suggest that Akita mice develop early features of diabetic kidney and heart disease but lack the more advanced features of these disease processes.

**Table 6:** Renal phenotype of male Akita mice by genetic background.

	C57BL/6	DBA/2	129/SvEv	FVB/NJ	C3H
Hyperglycemia	Sustained	Sustained	Sustained	Sustained	Sustained
Hypertension*	Yes	No	Yes	Yes	NR
Cardiac hypertrophy*	Yes	No	Yes	Yes	NR
Albuminuria* †	≈2–3 fold	≈3 fold	≈5 fold	>10 fold**	NR
GFR- Increased (early)*	≈0.3 fold	NR	≈0.25 fold	≈2 fold	NR
GFR - Decreased (late) †	Minimal‡	NR	NR	No	Minimal‡
Renal hypertrophy*	Yes	Yes	Yes	Yes	NR
Glomerular hypertrophy*	Yes	ND	Yes	Yes	NR
Mesangial expansion* †	Mild	ND	Mild	Moderate	Moderate
Arteriolar hylanosis†	NR	NR	NR	ND	NR
Interstitial fibrosis†	NR	NR	NR	ND	NR
GBM thickening†	NR	NR	NR	ND	NR
FP effacement	NR	NR	NR	ND	NR
Podocyte apoptosis	Yes (early)	NR	NR	Yes (early)	NR
Podocyte loss	Yes (late)	NR	NR	Yes (late)	NR

NR: not reported, ND: Not detected,

* Compared to strain matched non-diabetic controls,

† Animal models of diabetic complications consortium (ADMCC) criteria: >10-fold increase in albuminuria, >50% decrease in GFR over the lifetime of the animal, Advanced mesangial expansion, Arteriolar hylanosis, >50% increase in GBM thickness, Tubulointerstitial fibrosis,

** variable (see text for discussion),

‡ Increased BUN at 30–40 weeks of age, References: [3], [4], [5], [13], [30], [35], [48].

With regard to the current study, FVB/NJ Akita mice have robust albuminuria compared to other Akita strains but, similar to other Akita mice, the renal histopathological abnormalities are mild. These findings suggest a prominent dissociation between the functional and structural alterations in kidneys from FVB/NJ Akita mice. In the context of other published studies [5], [31], [32], these data are consistent with the notion that different genetic modifiers may separately affect functional and structural responses to the diabetic environment. Moreover, the robust albuminuria in FVB/NJ Akita mice compared to other Akita strains [4], [5] might provide an animal model that could be used to either identify genetic modifiers of urinary albumin excretion or test effects of antiproteinuric therapies. Indeed, Chua and coworkers [31] recently used linkage studies to identify a major locus influencing both albuminuria and renal pathology in a model of type 2 diabetes on the FVB/NJ background (FVB*^db/db^* mice).

In contrast to available renal functional and histopathological data in Akita mouse strains, there is limited published data on podocyte apoptosis and podocyte loss in Akita mice (Table 6). Similar to our findings in FVB/NJ Akita mice, Susztak et al [13] reported a significant increase in apoptosis early in the disease process in C57BL/6 Akita mice with podocyte depletion observed only in older animals. In the Susztak study [13], podocyte loss was dependent on reactive oxygen species (ROSs) generation because treatment with a NADPH oxidase inhibitor attenuated podocyte loss in this Akita mouse strain [13]. Similarly, we found [25] that ROSs generation promoted apoptosis of cultured podocytes through mechanisms that involve, in part, calcineurin (CN)-dependent induction of the enzyme cyclooxygenase 2 (COX2) and ROSs generation by COX2 enzymatic activity [25]. This observation appeared relevant to the in vivo situation because treatment with the CN inhibitor FK506 attenuated podocyte apoptosis in FVB/NJ Akita mice [25]. In support of a role for CN in this apoptotic response, studies by Abboud and coworkers found that CN activity was increased in rats following induction of hyperglycemia by streptozotocin (STZ) and treatment with pharmacological CN inhibitors reduced glomerular hypertrophy and extracellular matrix accumulation in the STZ model [33]. Taken together, these data suggest that CN and ROSs generation may play a key role in promoting podocyte loss and may contribute to glomerular hypertrophy and histopathological changes in diabetic kidney disease.

Given that hypertension and cardiac hypertrophy are commonly associated with diabetes mellitus and contribute to both cardiac and kidney damage in humans [34], we performed additional studies to investigate the cardiac phenotype of FVB/NJ Akita mice. Similar to diabetic humans, FVB/NJ Akita mice developed an increase in systolic BP, which was associated with cardiomegaly, LV systolic dysfunction and enhanced expression of molecular markers of cardiac hypertrophy including β-MHC as well as interstitial fibrosis and inflammatory cell infiltrates. Similarly, Hong and coworkers reported that Akita mice on the C57BL/6 background develop cardiac hypertrophy and echocardiographic evidence of LV dysfunction [35]. These data indicate that, in addition to studies of diabetic kidney disease, Akita mice on the FVB/NJ background may be useful for studying the mechanisms of cardiovascular injury in diabetes mellitus.

Lastly, in other studies using FVB/NJ mice, albumin excretion rates have been variable in diabetic animals [30], [31], [32], [36]. For example, Qi and coworkers [36] found that streptozotocin induced robust increases in 24-hour UAE in FVB/NJ mice but reported a poor correlation between UAE and ACR in these mice. Other investigators have reported more modest increases in ACR in FVB/NJ Akita mice [30]. There are several possible explanations for these discrepancies. First, it is possible that albuminuria in this strain may be subject to environmental influences as suggested by some investigators [37]. Secondly, in studies using streptozotocin, the non-specific toxicity of this agent [3] might alter the tubular handling of either albumin or creatinine. Finally, recent studies suggest that tubular creatinine secretion accounts for 35% to 50% of the excreted creatinine in urine of FVB/NJ mice [38], indicating that tubular secretion of creatinine is an important component of creatinine metabolism in this strain. In this scenario, high levels of urinary creatinine may blunt the ACR compared to 24-hour UAE measurements.

In summary, Akita mice on the FVB/NJ background developed cardiovascular complications and features of early stages of kidney injury in diabetic humans. FVB/NJ Akita mice developed sustained and durable hyperglycemia, which was associated with polyuria, albuminuria, renal and glomerular hypertrophy, glomerular mesangial expansion, podocyte apoptosis, a decrease in podocyte number and increased excretion of nephrin in urine. Urinary nephrin excretion was enhanced in Akita mice with albuminuria as well as early in the disease process with the onset of hyperglycemia and increased podocyte apoptosis but before the development of albuminuria. FVB/NJ Akita mice also had an increase in systolic BP and LV mass, which was accompanied by interstitial fibrosis and inflammatory cell infiltrates, echocardiographic evidence of LV systolic dysfunction, and enhanced expression of molecular markers of cardiac hypertrophy. Thus, our studies suggest that: 1. Akita mice on the FVB/NJ background have phenotypic features that may be useful for studying mechanisms of kidney and cardiac injury in diabetes mellitus, and 2. Enhanced urinary nephrin excretion is associated with kidney injury in FVB/NJ Akita mice and is detectable early in the disease process.

## Materials and Methods

### Ethics Statement

All animal care conformed to the National Institute of Health Guide for the Care and Use of Laboratory Animals, and was approved by the Institutional Animal Care and Use Committee at Duke University Medical Center, Durham, NC (Protocol Registry Number A007-10-01).

### Animals

Male heterozygous FVB/NJ-*Ins2* ^+/C96Y^ mice and wild-type FVB/NJ mice were purchased from The Jackson Laboratory (Bar Harbor, ME, USA) and then bred at the Genomic Science Research Building 2 at Duke University Medical Center, Durham, NC. Mice aged 4 weeks to 5 months old were used for experiments. To investigate the kidney phenotype mice were initially studied at 4, 8, 16 and 20 weeks of age and then sacrificed for tissue harvesting. A separate group of mice was also sacrificed for tissue harvesting at 30 weeks of age. Another group was studied at 4 weeks of age and then urine and tissue collected to measure albuminuria, podocyte apoptosis and podocyte number. To evaluate the cardiac phenotype, a fourth group of mice was studied at 10 weeks of age based on pilot studies suggesting that the elevation in systolic BP observed in Akita mice [4], [5] was prominent at this time point in FVB/NJ mice. Except for the fasting blood glucose measurements, all mice had free access to water and standard rodent chow throughout the study period (Lab Diet, Purina Mills, St. Louis, MO, USA). Insulin therapy was not required. Because of the mild hyperglycemia observed in diabetic, female mice, only male mice were studied in the experiments.

### Blood glucose measurements

Fasting blood glucose levels were initially measured at 4 weeks of age and then at 4-week intervals using an AlphaTRAK testing system (Abbott Laboratories, Inc., North Chicago, IL, USA). After fasting for 6 hours, approximately 2 µl of blood was collected directly on the testing strip for measurement after puncturing the lateral saphenous vein with a 25-gauge needle [39]. Free access to water was provided during the 6 hour fast.

### BP measurements

Systolic BP was measured using a computerized tail-cuff system (Hatteras Instruments, Cary, NC, USA) in conscious mice as previously described [40]. This technique has previously been shown to correlate closely with intra-arterial measurements [41].

### Echocardiographic analysis

Cardiac function and structure were evaluated in conscious mice at 10 weeks of age using echocardiography (ATL HDI 5000, General Electric Inc., Schenectady, NY, USA) as previously described [42]. 2-D guided M-mode and Doppler transthoracic images were obtained on conscious mice. Left ventricular end diastolic diameter (LVDd), left ventricular end systolic diameter (LVDs), interventricular septum width and posterior wall dimension were measured. Left ventricular fractional shortening was calculated as (LVDd – LVDs)/LVDd. Mean calculations were obtained from 3 consecutive cardiac cycles.

### Cardiac histopathology

Mice were sacrificed by an intraperitoneal injection of 250 mg/kg pentobarbital sodium (Ovation Pharmaceuticals, Deerfield, Il, USA) and exsanguination. Hearts were removed from a group of mice at 10 weeks of age and weighed. Hearts were then cut transversely, fixed in 10% formaldehyde, and embedded in paraffin. Sections were stained with hematoxylin and eosin (H&E) and Masson trichrome for examination by light microscopy. Interstitial fibrosis and inflammatory infiltrates were assessed using a semiquantitative scoring system from 0 to 3 as previously described with a scores of 1, 2 and 3 representing mild, moderate and severe histological abnormalities and a score of zero representing the absence of histological abnormalities.

### Urinary albumin, creatinine and nephrin measurements

Albuminuria was measured as previously described [43], using a kit from AssayPro, catalog number: EMS3201-1 (St. Charles, MO, USA), according to the manufacturer's instructions. Creatinine and nephrin levels in the urine were measured using respective kits from Exocell, Inc. (Philadelphia, PA, USA) according to directions of the manufacturer.

### GFR measurement

To assess GFR, fluorescein isothiocyanate (FITC)–inulin clearance was measured in conscious mice as described previously [5] using methods adapted from Breyer and coworkers [44].

### Renal histopathology

Mice were sacrificed by an intraperitoneal injection of 250 mg/kg pentobarbital sodium (Ovation Pharmaceuticals, Deerfield, Il, USA) and exsanguination. Their kidneys were then harvested for pathological examination using the following protocol. The left renal artery and vein were ligated, then the left kidney removed, decapsulated, and weighed. The abdominal aorta was then tied superiorly to the right renal artery. The right kidney was then perfused *in situ* with 4% paraformaldehyde trough a cannula inserted into abdominal aorta inferior to the origin of the right renal artery. The perfused right kidney was removed and embedded in paraffin. Sections were stained with H&E, Periodic acid-Schiff (PAS) and Masson trichrome for examination by light microscopy. Glomerular volume was determined from the mean cross-sectional area of 10 glomerular profiles on each animal using the method of Weibel-Gomez [45]. The extent of glomerular mesangial expansion was scored on a scale of 0–3 as described previously [4], [5]. GBM and proximal tubule basement membrane thickness were measured using transmission electron microscopy (TEM) by taking images in a sequential fashion from edge to edge. Basement membrane thickness was then measured using the orthogonal intercepts method [46]. Results are reported in nanometers (nm).

### Real-time RT-PCR

Total RNA was isolated from mouse hearts using TRIzol reagent (Invitrogen, Carlsbad, CA, USA) according to the manufacturer's protocol. The RNA was treated with RNAase free DNAase (Qiagen, Valencia, CA, USA) and then reverse-transcribed with Superscript reverse transcriptase (Invitrogen, Carlsbad, CA, USA) and oligo (dT) primers. Real-time quantitative PCR was performed using the ABI PRISM 7700 Sequence Detector System (Perkin-Elmer Applied Biosystems Division, Wellesley, MA, USA) and the universal SYBR Green PCR master Mix Kit (Perkin-Elmer Applied Biosystems Division, Wellesley, MA, USA). The amplification signals were analyzed with Perkin-Elmer ABI Prism 7700 Sequence detection software and were normalized to the endogenous β-actin mRNA level. The following sequences were used for the primers for mouse β-MHC, α-myosin heavy chain (α-MHC), atrial natriuretic peptide (ANP), and brain natriuretic peptide (BNP): β-MHC forward, 5′-GAG ACG GAG AAT GGC AAG AC-3′; reverse, 5′-AAG CGT AGC GCT CCT TGA G-3′; α-MHC forward, 5′-CCA CTG TGG TGC CTC GTT C-3′; reverse, 5′-GCG TCC GTC ATT CTG TCA CTC-3′; ANP forward, 5′-GCC ATA TTG GAG CAA ATC CT-3′; reverse, 5′-GCA GGT TCT TGA AAT CCA TCA-3′; BNP forward, 5′-AAG TCC TAG CCA GTC TCC AGA GCA-3′; reverse, 5′-AGA GCT GTC TCT GGG CCA TTT C-3′. The remaining primers were obtained from Applied Biosystems (Wellesley, MA).

### Measurement of podocyte apoptosis and podocyte number by immunohistochemistry

Mouse kidney cortex was embedded in Optimal Cutting Temperature compound and snap frozen in liquid nitrogen. Frozen sections were then fixed in acetone and air dried. Expression of Wilms tumor antigen-1 (WT-1) was assessed by indirect immunofluorescence using a rabbit polyclonal WT-1 antibody (Santa Cruz Biotechnology, Santa Cruz, CA, USA). Briefly, slides were treated with 1% Triton-X in phosphate buffered saline (PBS) and then blocked with 5% non-fat dry milk in PBS for 30 minutes. The WT-1 antibody was then added at a 1∶100 dilution in D-PBS with 5% non-fat dry milk. After 1 hour, slides were washed 3 times in D-PBS and then incubated for 1 hour with a fluoresceinated goat anti-rabbit antibody (Millipore, Bedford, MA, USA). After washing, terminal deoxynucleotidyl transferase dUTP nick end labeling (TUNEL) was performed using an ApoTag Red In Situ Apoptosis kit (Millipore, Bedford, MA, USA) according to the directions of the manufacturer. Slides were then washed 3 times and examined using a Nikon Eclipse TE2000-S fluorescent microscope. Podocyte number per glomerulus was determined by counting WT-1 labeled podocyte nuclei as previously described [25]. The number of apoptotic podocytes was determined by merging the WT-1 and TUNEL labeled images to detect apoptotic podocytes in all the glomeruli examined and the data was expressed as the number of apoptotic podocytes per glomerulus.

### Statistical analysis

Data are presented as the mean ± standard error of the mean (SEM) and statistical analyses were performed using the InStat computer program (GraphPad Sofware, Inc.). For comparisons of continuous variables, a test of normality was performed (Kolmogorov–Smirnov test) prior to assessing statistical significance using either a t-test (parametric) or Mann-Whitney test (nonparametric) [47] when comparing 2 groups. For comparisons between more than two groups, a test of normality was performed prior to assessing statistical significance using either: 1. A one way analysis of variance (ANOVA) followed by a Bonferonni multiple comparisons post test (parametric) [47], or 2. A Kruskal-Wallis test followed by a Dunn multiple comparisons post test (nonparametric) [47]. For comparisons of semiquantitative data, a Fisher's exact test was performed to determine statistical significance. P<0.05 was considered to represent a statistically significant difference.
